# Isolation and Characterization of 13 Microsatellite Loci from a Korean Endemic Species, *Sophora koreensis* (Fabaceae)

**DOI:** 10.3390/ijms130910765

**Published:** 2012-08-28

**Authors:** Ji-Yeon Lee, Dong-Hyuk Lee, Byoung-Hee Choi

**Affiliations:** 1 Department of Biological Sciences, Inha University, Incheon 402-751, Korea; E-Mails: leejiyeon@korea.kr (J.-Y.L.); dongh12345@hanmail.net (D.-H.L.); 2 Plant Resources Division, National Institute of Biological Resources, Incheon 404-708, Korea

**Keywords:** endemic species, genetic diversity, microsatellite, *Sophora koreensis*

## Abstract

To evaluate the population genetics structure as a means of devising conservation strategies, we developed microsatellite primers for *Sophora koreensis*, a narrowly endemic and endangered species in Korea. Thirteen polymorphic microsatellite markers were developed in Korean populations of *S. koreensis*. Genetic diversity was analyzed in 40 individuals from two populations. The number of alleles per locus ranged from 4 to 14, with observed and expected heterozygosities ranging from 0.200 to 1.000 and from 0.189 to 0.864, respectively. The microsatellite markers described here are valuable tools for the population genetics research of *S. koreensis*. They can be used to obtain information for creating suitable management strategies to conserve this endemic and endangered species.

## 1. Introduction

*Sophora koreensis* Nakai (Fabaceae) is a deciduous small shrub. Narrowly endemic, it is found at only a few locations in central Korea. Plants grow in dry soil in the understory of *Pinus* and *Quercus* forests. The shoots are interconnected by rhizomes, and propagation occurs both sexually (e.g., pollinated by the bumblebee *Bombus diversus diversus*) and vegetatively [1]. It is listed as an “Endangered Species” in the Rare Plant Data Book in Korea [2]. Based on its characteristics of four-winged legumes and vegetative propagation from long rhizomes, this species was previously separated from *Sophora* and categorized within the monotypic genus *Echinosophora* Nakai [3]. However, taxonomic studies based on pollen and petal morphologies and molecular evidence [4–6] have found that *Echinosophora* cannot be so clearly distinguished. Therefore, it is now treated again as a species within *Sophora* [7].

Numerous studies have indicated that narrowly endemic plants are susceptible to extinction for several reasons, one of the most important being habitat destruction [8]. In the case of *S. koreensis*, those populations are at risk from illegal plant collectors and projects such as trail or road construction. Despite the increasing demand for preservation and management plans, no suitable high-resolution genetic data have been available for this species. A population genetics study using dominant ISSR markers was performed to investigate the clonal and spatial genetic structures of *S. koreensis* [1]. However, those results were limited because the use of dominant markers does not allow scoring of genotypes. Heterozygosity is a more consistent measure, incorporating both total allelic diversity and the distribution of alleles among individuals that are affected by factors such as inbreeding and assortative mating [9]. Therefore, we have now developed a set of polymorphic and co-dominant microsatellite markers for *S. koreensis* that can serve as effective genetic markers. By conducting further assessments of its genetic diversity and population structure, we can strive toward establishing a strategy for its conservation.

## 2. Results and Discussion

We produced 13 polymorphic microsatellite loci that revealed clear and strong bands for each allele across two populations. Genotypic data were obtained for the 40 individuals from Mt. Bibong and Hanjeon-ri. Parameters for genetic diversity within each population are presented in Table 1. Overall, the alleles numbered 4 to 14 (average of 6.9); their observed and expected heterozygosities (*H*_o_ and *H*_e_) ranged from 0.200 to 1.000 and from 0.189 to 0.864, respectively (Table 1). Four of those 13 loci (Skor7, Skor10, Skor12, and Skor13) showed homozygotes or an excess of heterozygotes within the Mt. Bibong population, with significant deviations from the correction Hardy-Weinberg equilibrium (HWE) after a Bonferroni correction (*p* < 0.005; Table 1). No significant linkage disequilibrium (LD) was detected between locus pairs. These excesses might have been caused by a significantly high frequency of asexual reproduction that maintained heterozygotes or homozygotes over several generations.

## 3. Experimental Section

### 3.1. Isolation of Microsatellite Markers

Genomic DNA was extracted with a G-spin™ IIp Kit for plants (iNtRON, Seongnam, Korea). Microsatellites were developed according to the enrichment protocol [10], but with minor modifications. Briefly, genomic DNA was digested with *Mbo*I (Promega, Madison, WI, USA), and the resulting fragments were ligated to SAUL linkers [10] by using the T4 DNA ligase (Promega). Those fragments were then enriched for microsatellites with a cocktail comprising seven biotinylated probes [(AG)_15_, (AT)_15_, (AC)_15_, (ACG)_10_, (AGC)_10_, (CAA)_10_, and (ACAT)_7_] that were bound to streptavidin-coated magnetic beads (Promega). This enrichment process was performed twice. The fragments were then amplified and cloned using the PCR^®^ 2.1-TOPO^®^ vector (Invitrogen, Carlsbad, CA, USA). The size of the insert in 288 clones was evaluated by PCR, using the linker primer, and clones with 400- to 800-bp inserts were selected for sequencing. In all, 240 clones were subjected to double-stranded DNA sequencing that utilized BigDye Terminator version 3.1 and an ABI 3730xl sequencer (Applied Biosystems, Foster City, CA, USA). We culled the primers with short repeat regions or short flanking regions, or those that were close to the vector because they did not fit our criteria. Thirty-eight PCR primer pairs were designed with FastPCR version 5.4.51 software [11]. The M13 (-21) (5′-TGTAAAACGACGGCCAGT-3′) sequence-tag method was used to label those primers [12]. Of these 38, 17 failed to amplify, three had complex amplification, and five showed high frequencies of null alleles. Consequently, only 13 primer pairs could be successfully amplified within the expected size ranges. Loci characterizations and GenBank Accession Numbers for these 13 microsatellites are listed in Table 2.

### 3.2. Primer Validation

The microsatellites were characterized for samples from 30 individuals collected in South Korea from Mt. Bibong of Gangwon-do (38°06′30″N, 127°59′30″E) and 10 individuals collected in Hanjeon-ri of Gangwon-do (38°08′54″N, 128°00′59″E). Individuals were collected at >6-m intervals to avoid sampling a single genet more than once. DNA was extracted according to the method described above, and PCR was conducted with a GeneAmp^®^ PCR System 2700 Thermal Cycler (Applied Biosystems). Each reaction mixture contained 200 μM dNTPs (GeneCraft, Lüdinghausen, Germany), 1× PCR buffer with 1.5 mM MgCl_2_, 1 U of *Taq* DNA polymerase (TaKaRa, Seoul, Korea), 10 ng of DNA, and an appropriate concentration of primers in a total volume of 30 μL. The mixture also contained a 0.08 μM forward M13 (-21)-tagged primer, 0.3 μM reverse primer, and 0.3 μM M13 (-21)-labeled 6-FAM fluorescent dyes. PCR conditions included an initial denaturation at 94 °C for 2 min; followed by 38 cycles at 94 °C for 30 s, 52 °C for 45 s, and 72 °C for 1 min; with a final extension at 72 °C for 10 min. Fluorescently labeled PCR products were analyzed concurrently with the GeneScan™-500LIZ™ Size Standard (Applied Biosystems) on an ABI 3730xl sequencer, and sizes were determined with GENEMAPPER version 3.7 (Applied Biosystems). Diversity statistics, deviations from Hardy-Weinberg equilibrium (HWE), and linkage disequilibrium (LD) were estimated by GENEPOP version 4.0 software [13]. Null allele frequencies were calculated using MICRO-CHECKER version 2.2.3 [14].

## 4. Conclusions

The 13 microsatellite markers developed in this work are powerful genetic tools for studying populations and establishing effective strategies for management and conservation of *Sophora koreensis*. We also expect that these markers could provide information about the phylogeography of this species in Korea.

## Figures and Tables

**Table 1:** Results of initial primer screening in *Sophora koreensis. N*_a_, number of alleles; *H*_o_, observed heterozygosity; and *H*_e_, expected heterozygosity. *p*-Values for Hardy-Weinberg equilibrium (HWE) tests are given for each marker.

Locus	Mt. Bibong (*n* = 30)				Hanjeon-ri (*n* = 10)			
	
	*N*_a_	*H*_o_	*H*_e_	*p*-value	*N*_a_	*H*_o_	*H*_e_	*p*-value
Skor1	4	0.733	0.642	0.043	3	0.800	0.556	0.367
Skor2	8	0.768	0.783	0.036	4	0.600	0.611	0.213
Skor3	4	0.367	0.375	0.495	3	0.800	0.556	0.368
Skor4	4	0.700	0.544	0.092	3	0.800	0.644	0.834
Skor5	4	0.700	0.578	0.176	2	0.800	0.489	0.176
Skor6	9	0.667	0.602	0.378	5	1.000	0.744	0.004*
Skor7	7	0.900	0.807	0.000*	4	1.000	0.744	0.291
Skor8	7	0.867	0.795	0.230	5	0.800	0.800	0.010
Skor9	7	0.533	0.726	0.010	6	0.800	0.717	0.133
Skor10	13	0.933	0.864	0.000*	5	0.800	0.761	0.003*
Skor11	7	0.533	0.638	0.147	3	0.200	0.367	0.007
Skor12	4	0.267	0.571	0.000*	2	0.200	0.189	1.000
Skor13	6	0.467	0.699	0.000*	4	0.400	0.661	0.044

* Significant deviation from HWE after correction for multiple tests (*p* < 0.005).

**Table 2:** Characteristics of 13 microsatellite markers developed in *Sophora koreensis*. All forward primers were M13 (5′-TGTAAAACGACGGCCAGT-3′)-tailed at the 5′ end. Annealing temperature for each round of PCR was 52 °C.

Locus	Primer sequences (5′→3′)	Repeat motif	Size range (bp)	GenBank Accession No.
Skor1	F: ACTCAAAGCTGGAAGGGT R: TTCCGGTTGTTCGAGTCT	(TG)_13_	251–273	JX131650
Skor2	F: TGAACTACTACTCCGCTT R: ACCCCGTCAGCCAATACTTA	(AG)_14_	266–292	JX131651
Skor3	F: ATTCTGACTATTGGGCTAGTG R: ATCTGCTAAACCCTTGCTG	(GT)_20_	222–244	JX131652
Skor4	F: TCAATCACTATAAGGGTTCAG R: AAGTGTCAGGTGCATAAACC	(CAA)_12_	240–252	JX131653
Skor5	F: ATCAACTACTTCATGCGC R: ACAATCACTCCCAGCATAC	(TG)_14_	260–276	JX131654
Skor6	F: ACAAAAAGAAATGGTCGCG R: ACATGCCCCTCTGCCCCAAC	(CA)_21_(GA)_10_	242–294	JX131655
Skor7	F: TTGAAAAGTCCCACCTAGC R: TATCACCGCCAAGAACTTG	(GA)_26_	273–291	JX131656
Skor8	F: TCTAGGAGGAAATAGGGAG R: TAGACTCTTGGAACAAGAAC	(CA)_12_	285–297	JX131657
Skor9	F: TGGACACCCCTTATGGCTG R: ACCAAAGATTCCCGTGACAC	(AG)_19_	248–278	JX131658
Skor10	F: TTGGGATAGAGTCACGTCC R: ACCGAACATGTCTCATTACC	(GT)_11_(GA)_15_	222–270	JX131659
Skor11	F: TAAAGTGCATGTTCCCAG R: TGCTGAACCCTTAGAGAGG	(CA)_10_	256–296	JX131660
Skor12	F: TGAACTTAGCATTCCATTC R: AAGCGGTTAGACTTTCAGC	(GA)_10_	253–267	JX131661
Skor13	F: TTCAATGGGTTATTGGTCC R: ATGTATCTTCTGGGACTCG	(TG)_14_	238–250	JX131662
